# The psycho-sensory wake drive—a power source for power naps and other common sleep-wake phenomena: a hypothesis

**DOI:** 10.1007/s11325-017-1505-6

**Published:** 2017-04-29

**Authors:** Peter T. George

**Affiliations:** 1649 Kalakaua Ave, Suite 204, Honolulu, HI 96826 USA

**Keywords:** Sleepiness, Multiple sleep latency test, Alertness, Ascending activating system, Two-process model of sleep-wake regulation

## Abstract

Power naps are extensively practiced worldwide and there exists ample documentation of their efficacy in reversing daytime sleepiness. The source of their efficacy, however, as well as the cause and manifestation of many other common sleep-wake phenomena, cannot be entirely explained by the most commonly accepted model of sleep-wake regulation, the two-process model of Borbély, which considers the drives of the circadian and homeostatic sleep processes only. When considering the causes and manifestations of these unexplained phenomena, there appears to be evidence of a wake-promoting drive that is independent of the circadian oscillator indicated in the two-process model of sleep-wake regulation. Although this posited secondary wake drive, herein referred to as the psycho-sensory wake drive, is always active during the awake state, its strength unpredictably varies during a normal day and, therefore, cannot be incorporated into the prevalent two-process model by any current mathematical formula. However, a supplemental graphic model superimposing it on the drives of Process S and Process C can provides plausible and parsimonious explanations for many otherwise unexplainable sleep-wake phenomena and enables rational guidelines for their effective practical management.

## Introduction

In 1998, James B. Maas coined the term “power nap” to “encourage institutionalization of naps at work” [1]. Since that time, substantial additional documentation indicating the efficacy of brief naps in the maintenance of on-the-job performance has been accumulated [2]. Although it is well documented that brief naps are more effective in the apparent reversal of sleepiness than longer ones [3–5], the scientific community has been unable to fully explain this paradox. Sleep researchers have known for more than a decade that longer naps are more likely to result in sleep inertia—a period of disorientation, confusion, and sleepiness that results after awakening from a deeper sleep stage [6]. Sleep inertia may partially explain why longer naps are not as refreshing as shorter ones, but if short naps do not reach a deep stage of sleep, then why are they reinvigorating?

The two-process model of sleep-wake regulation [7] is the model most commonly referred to in sleep science literature. For more than three decades, this model has continued to accurately predict and logically explain many aspects of sleep timing via the interactions of the drives in the homeostatic sleep process (Process S) and the circadian sleep process (Process C). However, although this model has been periodically refined [8–10], many intriguing questions remain unanswered [11]. Not only can this two-process model not identify the source of power in the power nap but it also cannot explain how most people can lie down and fall asleep within 15 min at any time of day [12] or how an alarm can awaken a person in the middle of the night. It also does not explain how a college student who has difficulty staying awake during what he or she considers a dull afternoon lecture can later party well past midnight—a time when both processes predict that the student would be considerably sleepier—without an intervening nap. Furthermore, the two-process model cannot explain how an individual can score as sleepy on a standard objective test of sleepiness, but score as alert on a standard objective test of alertness on the same day [13]. The answers to these questions may have applications to alertness problems encountered by many populations, including night shift workers, transmeridian travelers, drowsy patients with sleep apnea, and bored college students.

In addition to the two-process model’s inability to answer the intriguing questions of how it is possible for a 10- to 20-min nap [3–5, 14] or a 40-min meditation session [15] to immediately elevate an individual’s vigilance, it does not shed much light on the causes of many cases of insomnia [16, 17]. Furthermore, the two-process model does not provide clues regarding how a 6-min nap can enhance declarative memory performance [18]. Explanations addressing the above questions may be of value to students, practitioners, or researchers involved in sleep-wake problems.

When considering the causes of the above-unexplained phenomena, there appears to be evidence of a wake-promoting drive that is independent of the circadian oscillator indicated in the two-process model of sleep-wake regulation. The aims of this article, therefore, are to consider apparent evidence for the existence of this drive and to hypothesize how, when combined with the drives in the two-process model, this drive may reflect a source of efficacy in power naps and other common but unheeded sleep-wake phenomena and thus enable their more practical and effective management.

## Background

In the 1970s, the two main theories concerning sleep regulation and timing were the restorative and adaptive theories [19]. The restorative theory indicates that sleep enables the mind and body to homeostatically recuperate from the wear and tear that accumulates during daily activity. The adaptive theory proposes that sleep occurs during specific temporal periods in different species as a behavior that enhances survival [20]. In the early 1980s, Alexander Borbély elegantly combined these theories into the two-process model of sleep regulation [7], wherein the restorative theory became Process S to indicate the homeostatic accumulation and depletion of sleep propensity, and the adaptive theory became Process C to indicate the circadian temporal propensity of sleep.

**Fig. 1:**
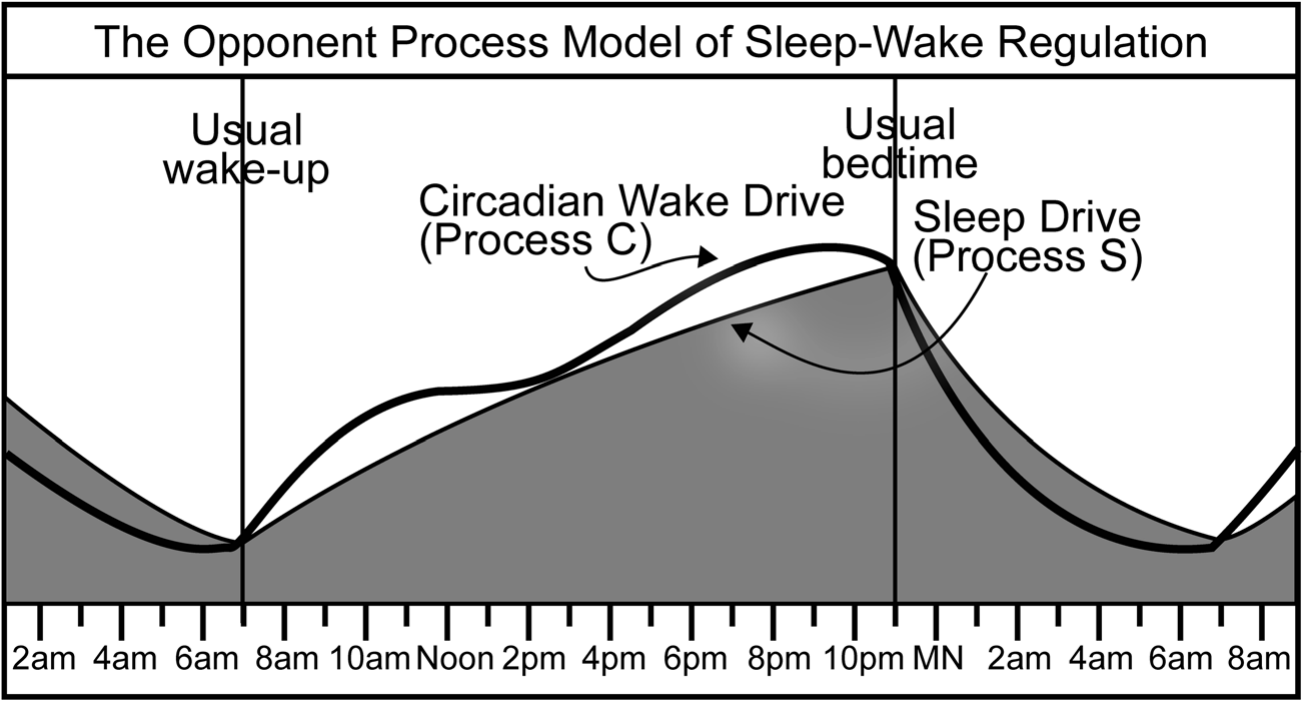
An opponent process model shows how a circadian wake drive (Process C) and a homeostatic sleep drive (Process S) may interact to result in the daily sleep-wake cycle. The wake drive, the *heavy black line*, is programmed by the suprachiasmatic nucleus to gradually undulate upward during the day and rapidly fall during the night. The sleep drive, the *dark gray area*, always rises while awake and always falls while asleep. When the wake drive is on top, the subject is awake, and when the sleep drive is on top, the subject is asleep

In the original model, both processes regulate the propensity and maintenance of sleep only. Process S promotes sleep and Process C gates its onset and termination. In the early 1990s, Dale Edgar and colleagues presented research indicating that the primary function of Process C is not to gate nightly sleep, but rather to promote subjective day wakefulness to oppose sleep propensity [21]. Based on their research, Edgar et al. proposed the opponent process model of sleep regulation. Figure 1 graphically depicts how these two processes might oppose one another to regulate the daily sleep-wake cycle [22].

**Fig. 2:**
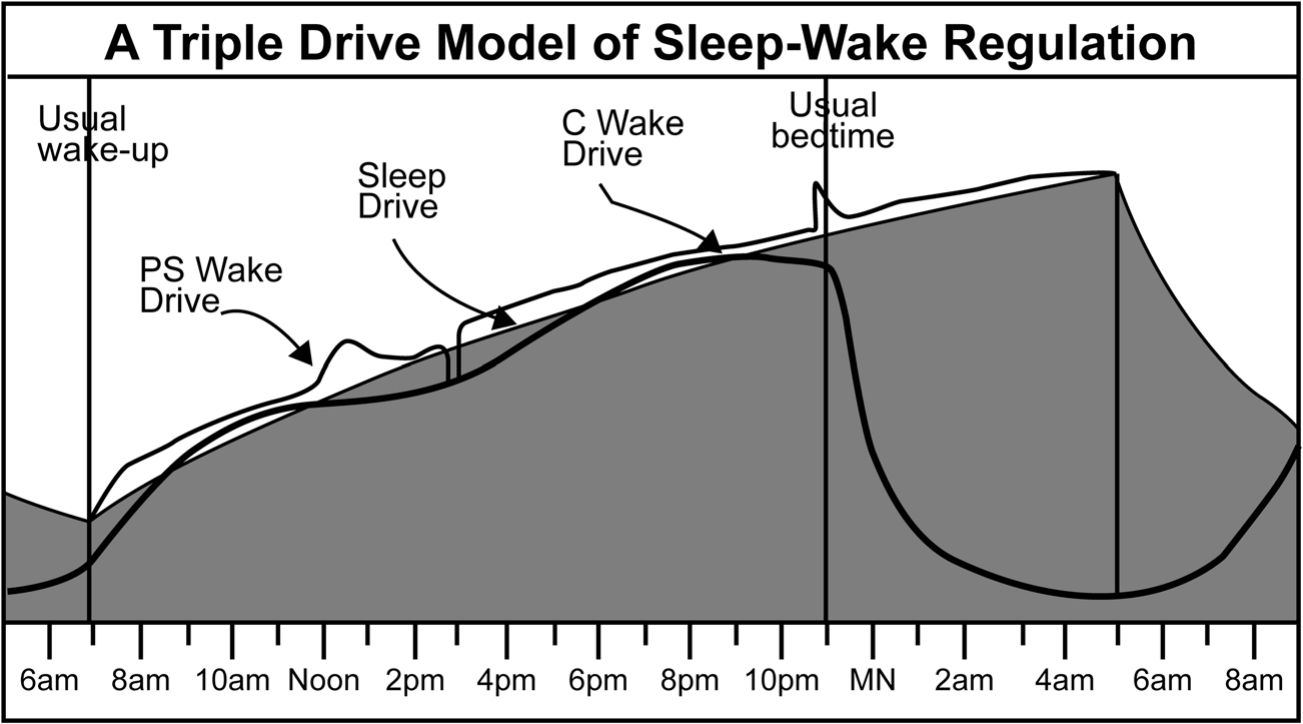
A triple-drive model graphically indicates how the PS wake drive may be added transitorily on top of the C wake drive to enable and enhance wakefulness when previous inadequate sleep causes the S drive to rise above it. An alarm is needed to initiate the PS wake drive at 7:00 a.m., while stimulating events lead to peaks in this drive. When an individual naps at 2:45 p.m., voluntarily stays awake beyond his usual bedtime, and falls asleep at 5:00 a.m., the PS wake drive disappears. Curve heights are selected only to illustrate the concept of the model and cannot be used for mathematical calculations

Also in the early 1990s, Murray Johns suggested that the model of sleep-wake regulation should contain a secondary wake drive to account for behavioral influences on wakefulness in addition to the circadian wake process [23, 24]. Johns noted that the additive inputs to the central nervous system from postural muscles, joints, and other proprioceptive nerve tracts, as well as those from visual and other exteroceptive and enteroceptive inputs, drive wakefulness. Johns recently proposed calling this drive Process A to indicate that it is driven by the afferent nervous system [25]. However, given evidence that this process includes emotional and cognitive inputs [26] in addition to sensory inputs and is also largely voluntary, the term psycho-sensory (PS) is a more inclusive reference to the source of this drive. Adding this expanded version of Johns’ secondary wake drive (PS wake drive) to the schematic version of the opponent process model [22] presented in Fig. 1 creates a transitory model that may be called a graphic triple-drive model of sleep-wake regulation (Fig. 2).

It is common knowledge that proprioceptive, exteroceptive, enteroceptive, cognitive, and emotional stimulation can initiate and maintain wakefulness. Impulses initiated by these stimuli traverse the central nervous system (CNS) to activate the ascending arousal system (AAS), which comprises several brain structures within the brainstem, thalamus, hypothalamus, and basal forebrain that release multiple monoamine neurotransmitters and a cholinergic neurotransmitter, all of which drive wakefulness [27]. Activation of the AAS nuclei, in turn, promotes arousal within the thalamus and cerebral cortex, as indicated by electroencephalography (EEG) desynchronization [28]. This EEG desynchronization appears to be the manifestation of the PS wake drive.

Unlike input to the C wake drive, input to the PS wake drive can be both voluntary and reactive to its surroundings. Although its output is transitory and cannot be predicted or calculated by any existing mathematical formula, its addition to a graphic version of the two-process model creates a novel model that enables plausible explanations of many common sleep-wake phenomena that cannot be described by the two-process model in its current form.

This triple-drive model posits that the C wake drive alone is often insufficient to initiate and maintain wakefulness, especially when sleep debt exists, as indicated in Fig. 2 (the sleep drive in this example is above the C wake drive at the time of usual wake-up). There is a greater need for PS wake drive supplementation in the afternoon during the midday dip in the C wake drive, but less need for the PS wake drive in the mid-morning and mid-evening during the two daily crests in the C wake drive [29].

The PS wake drive appears to supplement the C wake drive to oppose the S drive as needed. The timing and strength of the C wake drive do not vary from day to day, but the timing and strength of the PS wake drive immeasurably vary during a normal day. Although the PS wake drive can be modified by voluntary control, it automatically turns on and off when awakening and falling asleep, respectively, representing various sense receptors that automatically transmit impulses to the AAS through collaterals after awakening but cease after falling asleep [30, 31].

## Explanation of common sleep-wake phenomena

Figure 2 depicts hypothetical PS wake drive responses to various stimuli in a normal subject, who is hereafter referred to as Joe. Prior to 7:00 a.m., Joe, who has a sleep debt, is asleep and, therefore, has no PS wake drive. At 7:00 a.m., an acoustical alarm initiates neural impulses that traverse the subject’s AAS to reach his cerebral cortex, which immediately creates a PS wake drive sufficient to awaken him [30, 31]. This wake drive is added to the C wake drive to create the total wake drive. There is no known way to determine the absolute heights of the curves that represent these drives. The relative heights of the curves in Fig. 2 were arbitrarily selected by the author to illustrate the conceptual model and cannot be used for mathematical calculations.

In Fig. 2, Joe’s alertness is relatively constant from 8:00 am until approximately noon. The generation of his PS wake drive (measured vertically from the top of the C wake drive) declines before noon, although his level of alertness remains constant due to the rise of the first daily crest of his C wake drive [29]. Joe’s PS wake drive spikes around noon due to an exciting lunch date. After lunch, Joe returns to his office and while reading dull reports, his PS wake drive declines to near his sleep drive. He decides to take a 15-min nap. To facilitate sleep onset, he attempts to minimize PS stimulation by putting on an eye mask and assuming a recumbent posture. Sleep onset eliminates the subject’s PS wake drive and an alarm is required to reinitiate it. The greater height of the PS wake drive immediately following Joe’s nap indicates his significantly increased alertness [32]. The subject’s PS wake drive remains well above his sleep drive until his C wake drive reaches its second daily crest [29]. As Joe prepares for his usual 11:00 p.m. bedtime, he is startled by a phone call from his employer informing him that he must complete and email a report that was needed for an emergency meeting the following morning. His employer also informs Joe that he need not report to work the next day until noon. Joe falls asleep at 5:00 a.m., but spontaneously wakes at 9:00 a.m. when his C wake drive rises above his sleep drive.

As indicated above, the PS wake drive is created and sustained by involuntary or voluntary input into the AAS. Acknowledging its origination and presence can help explain many common phenomena not explained by knowledge of an individual’s current sleep debt or circadian position (e.g., the erratic fluctuations of Joe’s alertness in Fig. 2). It is commonly acknowledged that we have significant control over our sleep-wake status. Most people can voluntarily reduce their PS wake drive input sufficiently to fall asleep within 15 min at any time [12]. Conversely, voluntary adjustment of the PS wake drive magnitude through additive AAS input enables us to counter the diminution of our C wake drive to maintain wakefulness in situations involving night shift work, transmeridian travel, or post-midnight festivities. It is common knowledge that insomnia is often caused by the inability to control internally or externally generated AAS input. The magnitude of the PS wake drive can far exceed that of the C wake drive at its peak (e.g., in an individual who is awake at 4:30 a.m. and has not slept since 7:00 a.m. the previous day [Fig. 2]). One 18-year-old boy was able to generate enough PS wake drive to enable him to successfully compete in miniature mechanical baseball games while remaining awake continuously for 11 days [33, 34].

## Sleep tests, PS wake drive, and other arousal forces

The multiple sleep latency test (MSLT) is a standard physiological measure of sleepiness [12]. This test assumes that sleepiness is the tendency to fall asleep and that its severity can be determined by measuring how quickly one can fall asleep. Its developers sought to measure “physiological sleepiness,” which is determined only by the magnitude of the sleep debt (S drive) resulting from sleep deprivation rather than “manifest sleepiness,” which is in part determined by concurrent “alerting factors” [12]. The developers of this test therefore attempted to minimize psycho-sensory stimulation, which they believed “masked” the physiological sleep tendency. To achieve this, they tested subjects in a constant routine that requires lying in bed in a dark quiet room after being given the following instructions: “close your eyes and try to fall asleep” [35]. Although this routine reduces psycho-sensory stimulation, varying amounts of stimulation are unavoidable (e.g., from sensory awareness of the electrodes attached to obtain EEG data or from psychic reactions to the procedure).

The MSLT was quickly incorporated into clinical practice [36]. However, some practitioners found that this test lacked sensitivity in hypersomnolent patients due to its floor effect [37]. Partially in response to this criticism, the maintenance of wakefulness test (MWT) was developed in 1982 via modifications to the MSLT [38]. These modifications involved increasing the amount of psycho-sensory stimulation the subject was exposed to during testing by requiring the subject to sit upright rather than lie in a bed. In addition, the test was carried out in a dimly lit room rather than a dark room, with instructions to stay awake rather than to try to fall asleep.

While the MSLT is considered an objective test of sleepiness and the MWT [38] is widely considered an objective measure of alertness, both aim to measure the subject’s position on the sleepiness-alertness continuum [39]. Therefore, if one scores as sleepy or alert on one test, then he or she should have the same result for the other test. Although results from the two tests are normally consistent, when comparing results of these two tests from the same subjects on the same day, some appear very sleepy when tested on the MSLT but very alert on the MWT [13]. These discrepant results indicate the existence of at least one factor in addition to Process S and Process C that influences daytime sleepiness-alertness. The hypothesis of this study posits that because consideration of the drive in Process C is eliminated due to the multiple recordings of these tests, which average out any temporal influence, the PS wake drive is the only major force that can account for this discrepancy, and that some individuals can control the levels of their PS wake drive through psychic stimulation or suppression better than others can [40, 41].

There is no known method to determine to what degree the results of either of these two tests are due to the S drive or the PS wake drive. The MSLT attempts to minimize the PS wake drive and, therefore, is a better measure of the S drive. The MWT invokes the PS wake drive by including more sensory and psychic stimulation. Therefore, the MWT tests the subject’s ability to control his or her PS wake drive while simultaneously testing the level of the subject’s S drive. Consequently, the MWT is not a reliable test of either the PS wake drive or the S drive. However, the MWT is a better test of sleepiness (i.e., the propensity to fall asleep) than the MSLT if it is assumed that the PS wake drive is the major opponent of the S drive in these tests.

The Epworth Sleepiness Scale [23, 42] may provide a more accurate measure of sleepiness (propensity to fall asleep) because it attempts to fully integrate the influence of the PS wake drive when it asks subjects to rate their sleepiness in common situations of known somnificity [43]. This term, coined by Murray Johns, is defined as “the general characteristic of a posture, activity, and situation that reflects its capacity to facilitate sleep onset in the majority of subjects” [25]. In other words, somnificity is the degree to which a posture, activity, or situation reduces the PS wake drive, that is, the combined exogenous and endogenous inputs into the AAS. When subjects assign numerical indications (0–3) of their likelihood to doze off or fall asleep in each of the eight common daily situations with varying somnificity, the total score provides an average sleep propensity.

From the information presented above, it is apparent that arousal forces in addition to those in Process C influence sleep-wake regulation. This article submits that the PS wake drive described herein is one of these arousal forces. According to the model of sleepiness described by De Valck and Cluydts, which suggests that arousal forces may be classified as state (acute) or trait (chronic) [11], the PS wake drive is a state due to its transient nature. As defined in this article, the PS wake drive originates from psycho-sensory stimuli that send impulses through the AAS to the cerebral cortex that increase alertness as evidenced by EEG desynchronization. There is evidence of arousal forces—in addition to Process C—that influence sleep-wake regulation and that work outside of this definition. These include forces that produce constant hyperarousal resulting in insomnia [44, 45]. The existence of these forces is acknowledged, but not discussed, here because they are not essential to the operation of the PS wake drive. A recent review by Bakotic and Radosevic-Vidacek provides a comprehensive discussion of the various arousal forces [46].

## How brief naps may enhance alertness

According to the two-process model of sleep-wake regulation, excess Process S is the only cause of daytime sleepiness, and sleep—specifically, sleep with slow-wave activity (SWA) —is the only means to reduce Process S. The deeper the stage of sleep is, the more rapidly Process S is reduced [47]. Only a negligible amount of SWA normally occurs within 15 min of sleep onset [48]. In fact, a number of studies have documented that a significant decrease in daytime sleepiness occurs following naps of 10 or less minutes [32]. There thus appears that there is no means by which the two-process model can explain the source of benefits from brief naps. In addition, brief naps result in rejuvenated awakenings, not only in sleep-deprived subjects with larger than normal sleep debts but also in those who have slept a normal amount the previous night [3, 4].

Lovato and Lack [32] have suggested that the rejuvenation experienced immediately after a brief nap may be attributable to the process of sleep onset (Process O), which involves the sleep-switch mechanism described by Saper et al. [49]. This switch, which toggles sleep and wakefulness on and off, is a joint property of sleep-active nuclei in the ventrolateral preoptic area of the hypothalamus (VLPO) and wake-active nuclei in the AAS. Process O derives its efficacy from the mutual inhibition of these nuclei, so that only one state—asleep or awake—can be active at one time. That is, the cerebral cortex can only receive impulses from one of these two sleep-wake centers at a given time [49]. The two-process model, as portrayed in Fig. 1, does not consider any input from the PS wake drive. It only considers input from the sleep process, which grows incrementally and does not fluctuate during the day, and the circadian process, which fluctuates on a predetermined course. The triple-drive model, as portrayed in Fig. 2, describes the state change resulting from the toggling of the sleep switch, which turns off the PS wake drive when the subject naps at 2:45 p.m.

The Lovato and Lack hypothesis that Process O enhances alertness [32] indicates that the wake-active nuclei of the AAS lose some of their capacity to transmit impulses to the cortex after individuals have been awake for 2–3 h. However, this partial loss of excitability may be regained via Process O as follows. When sleep onset occurs, the sleep-active neurons of the VLPO are switched on (activated) and the wake-active neurons of the AAS are automatically switched off (inhibited). This causes a complete cessation of AAS activity, which allows restoration of the original excitability in its nuclei to occur within approximately 10 min [32]. This renewed excitability of the AAS allows it to then send more powerful impulses to the cortex, as indicated by EEG desynchronization, which enhances subjective alertness and performance on tasks requiring vigilance. This decrease and regeneration of AAS excitability, as viewed through the prism of the triple-drive model, may be interpreted as a decrease and increase, respectively, in the force of the PS wake drive. This process of relatively slow depletion and rapid restoration of force—similar to that wherein a battery operates at nearly full force for hours but can be fully recharged in minutes—is not a novel physiologic concept [50, 51].

In 2008, Lahl et al. [18] reported that a 6-min nap significantly enhances declarative memory performance. Subjects who did not nap recalled slightly fewer than seven previously memorized words. After the same time period and a 6- or 35-min nap, the subjects recalled slightly more than eight or nine words, respectively. The benefits of the longer nap did not correlate with time spent in slow-wave sleep (i.e., Process S). The authors speculated that the mere onset of sleep may in some unexplained manner “… initiate active processes of memory consolidation which—once triggered—remain effective even if sleep is terminated shortly thereafter.” However, the triple-drive model, as presented above, suggests that a more likely cause of superior post-nap test scores is greater alertness due to the “recharging” of the AAS. Lovato and Lack [32] note that it takes 7–10 min of sleep for the AAS to regain its full excitability. A rate curve of this return of excitability needs to be established. However, because the superior results of the longer nap could not be attributed to more slow-wave sleep, it appears that if the nappers were tested after a little more than 10 min of sleep, the results would have been approximately the same as those observed after 35 min of sleep.

Kaul et al. [15] recently showed that the rejuvenating effect experienced after brief naps can also be experienced after 40-min sessions of simple eyes-closed concentrative meditation in subjects with no prior meditative experience. These researchers detected no sleep in their subjects. Therefore, no sleep-switch flipping occurred, and Process O did not appear to be necessary. Instead, psycho-sensory stimulation into the AAS appeared to have been reduced in these subjects for 40 min. Although the AAS has been labeled as a wake center [32], it is not likely to be the generator of the PS wake drive. Rather, the AAS appears to be the conduit or gate that modifies the input to the cortex. The 40-min period of reduced activity appears to have allowed rejuvenation of this wake center, much like the rejuvenation of a runner after slowing down to a leisurely walk. Supporting this conjecture is the fact that no claims for an immediate pre-test to post-test vigilance improvement of the magnitude demonstrated by Kaul et al. [15] can be found in the literature for the much-studied 20-min transcendental meditation. Further research on various meditation types and lengths is required.

**Fig. 3:**
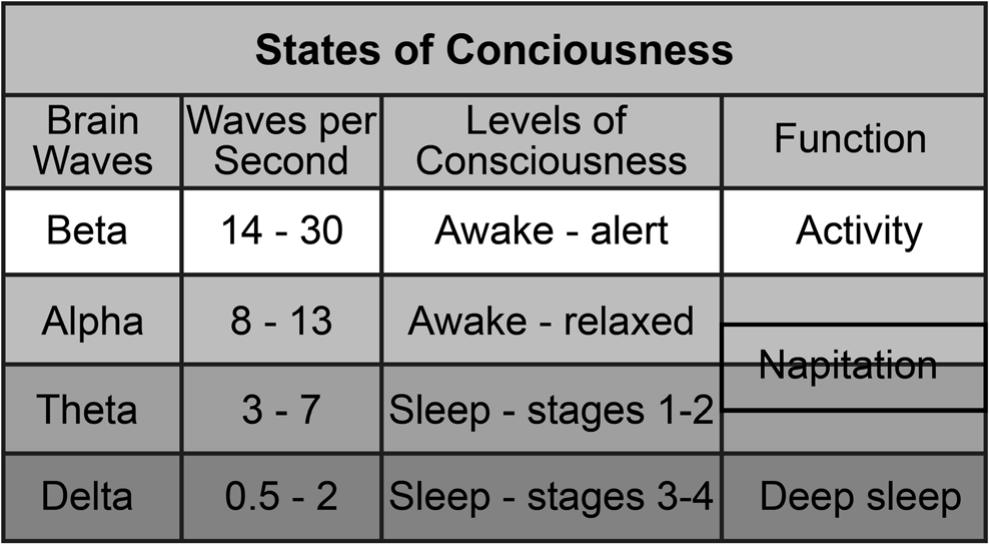
Napitation occurs in the state of consciousness between activity and deep sleep when the brain produces alpha and theta waves of 5 to 10 Hz. This state may be reached by a brief nap or meditation

The term “napitation” is introduced here to indicate a protocol containing either a brief nap or meditation session that results in immediate restoration of alertness. Both procedures reduce psycho-sensory input into the AAS, resulting in less cortical stimulation and slower brain wave frequencies, as measured using EEG. Figure 3 shows the brain wave frequency ranges of sleep-wake states from alert to deep sleep [52]. Brain wave frequencies between approximately 5 and 10 Hz [53] have been labeled as the napitation range on this chart because they appear to be the frequencies that must be maintained during a nap or meditation session to result in greater vigilance upon awakening.

Slower brain wave speeds during napitation appear to allow more rapid restoration of AAS excitability. However, subjects in both procedures have floors below which they must not descend. The brain wave frequencies of meditators cannot fall below approximately 7–8 Hz without falling asleep [52, 53], and those of nappers cannot fall below approximately 4–5 Hz without incurring sleep inertia [6]. The meditators in the Kaul et al. [15] study appeared to maintain their brain wave frequencies within the narrow napitation range above the frequency required to enter stage 1 sleep by concentrating on their breathing to block stimulating thoughts and appeared to prevent sleep by maintaining an upright kneeling posture to ensure continuous proprioceptive stimulation. Power napping subjects must prevent their brain wave frequencies from descending to levels that result in sleep inertia by setting an alarm that signals after 10 min of sleep, plus the estimated latency period to sleep onset.

## Conclusions

Voluntary and involuntary sensory, cognitive, and emotional stimuli in the daily activities of any normal subject promote wakefulness and oppose sleep. These exogenous and endogenous stimuli traverse the CNS via the AAS to the cerebral cortex, where they combine to form the PS wake drive, which supplements the circadian wake drive originating in the suprachiasmatic nucleus. Although there currently is no available mathematical formula to incorporate the PS drive into the most commonly used two-process model of sleep-wake regulation, its visualization in a graphic version of the model enables parsimonious plausible explanations of many common sleep-wake phenomena that cannot be explained by the two-process model in its current form.

The reversal of sleepiness following 10 min of sleep or 40 min of meditation appears to be due to reductions in AAS activity, which allow a return of excitability to AAS nuclei and enable greater cerebral cortex stimulation resulting in a stronger PS wake drive upon awakening.
